# A nonmagnetic micropositioner for displacement measurement validation in the MRI environment

**DOI:** 10.1007/s10334-026-01341-y

**Published:** 2026-04-21

**Authors:** William Ryan Willoughby, Mark Bolding

**Affiliations:** Department of Radiology, Heersink School of Medicine, The University of Alabama at Birmingham, Birmingham, AL USA

**Keywords:** Magnetic resonance imaging, Bias, Signal-to-noise ratio, Regression analysis

## Abstract

**Objective:**

The purpose of this study was to implement a nonmagnetic micropositioner in the MRI environment to validate displacement estimates of magnetic resonance acoustic radiation force imaging (MR-ARFI).

**Methods:**

The micropositioner consisted of a stage driven by a piezoelectric stepper motor in closed-loop operation with an optical quadrature encoder. A 100-gram agar gel phantom was prepared, and three MR-ARFI pulse sequences were used to generate displacement maps. MR-ARFI measured displacements were compared to ground truth data from the optical encoder.

**Results:**

The micropositioner demonstrated consistent performance with positioning times of 1.6±0.4 ms for extension and 2.2±0.2 ms for return to baseline position. The micropositioner decreased the signal-to-noise ratio of magnitude images due to increased electronic noise. Linear regression analysis showed that displacement measurements were highly linear with $R^2 \ge 0.98$ but exhibited scaling biases that may have been due to the experimental setup.

**Conclusion:**

The proposed instrument can potentially improve the accuracy and precision of MR-ARFI-based applications, including focused ultrasound dosimetry and mechanical biomarker imaging.

## Introduction

Measurement of microscopic tissue deformation in response to applied stress is the basis for medical imaging of mechanical tissue properties, or elastography. Elastography techniques using acoustic radiation force impulses (ARFI) as the applied stress have been developed for ultrasound and magnetic resonance (MR) imaging modalities [1]. MR acoustic radiation force imaging (MR-ARFI) estimates the quasistatic deformation of tissue induced by ARFI and is used for real-time targeting and evaluation of focused ultrasound (FUS) treatments [2]. Like the ARFI ultrasound imaging technique, ultrasound bursts are applied and a volumetric force is induced as the waves are attenuated and momentum is transferred to tissues [3]. Ultrasound ARFI uses cross-correlation analysis of radiofrequency ultrasound echoes to generate maps of tissue stiffness [4]. MR-ARFI is a phase-contrast technique that uses motion-encoding gradient (MEG) pulses to encode the micrometer-scale displacement of water protons into the phase of free induction decay (FID), gradient-recalled echo (GRE), or spin echo (SE) magnetic resonance signals.

In MR-ARFI, pairs of images are acquired, either with MEGs of opposite polarity or with one image acquired without ARFI, effectively doubling the acquisition time of a particular pulse sequence. In the case of baseline imaging without ARFI, the phase shift $\Delta \phi$ accrued by an ensemble of protons which is displaced from its initial position by *u* then subjected to a constant magnetic field gradient with amplitude *G* and duration τ is [5]

1 $$\begin{aligned} \Delta \phi = \gamma G \tau u \end{aligned}$$

where γ= 2.6752 $\times 10^{8}$ rad/(s T) is the proton gyromagnetic ratio. This phase shift is relative to the phase of a stationary ensemble resonating at the same frequency. Equation (1) holds only for the ideal case in which the ensemble remains motionless and the magnetic field gradient is completely static during the encoding period. Despite the idealization, this formula is frequently used to determine the displacement from the MR phase shift. The complex subtraction of image pairs reduces the systematic background phase variations that result from inhomogeneities in the main magnetic field $B_0$. If image pairs are acquired using two ARFI pulses and opposing MEG directions, the sensitivity is effectively doubled, but the ultrasound dose is also doubled, which may not be desired.

MR-ARFI has become increasingly useful in the clinical setting, where the trade-off between diagnostic power and imaging time must be carefully considered [6]. The original implementation of MR-ARFI used a line scan spin echo pulse sequence [7]. Column-by-column single-shot acquisition was selected to minimize artifacts from motion, but long acquisition times associated with this type of scan limited the field of view and spatial resolution. Echo planar imaging (EPI) and spiral readouts have reduced typical acquisition times for two-dimensional images to less than 1 s [8–10]. A balanced steady-state free precession (bSSFP) sequence improved sensitivity and allowed for targeting with ultrasound pulses as short as 1.45 ms [11]. MR-ARFI pulse sequence variants have been developed to perform shear wave elastography (SWE), mapping shear wave speed and estimating stiffness based on displacement maps acquired at different time points [12, 13].

To optimize MR-ARFI pulse sequences for imaging quantitative biomarkers, such as shear modulus and Young’s modulus, for measuring ultrasound dose in HIFU treatments, and for evaluating the bioeffects of HIFU interventions in situ, instrumentation is needed to validate the displacement measurements of MR-ARFI.

Various techniques have been used to independently measure displacements in the MRI environment. Due to the need to reduce the acoustic output of MRI scanners during image acquisition, studies have used pressure transducers and laser Doppler vibrometry (LDV) to measure mechanical vibrations on scanner components [14]. Nwotchouang et al. developed a device to mimic the pulsatile motion of brain tissue and used LDV to determine the accuracy of a displacement-encoding with stimulated echoes (DENSE) MRI pulse sequence [15]. Their device used plastic shafts to deliver a periodic mechanical force from a high-torque motor to one side of the phantom. They found that DENSE MRI agreed with LDV measurements with about 5% displacement error at displacements down to ${220}\,{\upmu }\text {m}$. Brinker et al. compared magnetic resonance elastography (MRE) acquisitions to scanning optical LDV data and found good correlations between the two methods on the surface of elastic materials [16].

The purpose of this work was to implement a computerized micropositioner in the MRI environment to validate MR-ARFI micrometer-scale displacement measurements. To the best of our knowledge, this is the first time an instrument like this has been used to evaluate the accuracy of MRI-based displacement measurements. This is also the first study to compare MR-ARFI displacement measurements with independently acquired measurements.

Several design constraints had to be considered for instrument functionality and MRI compatibility. The micropositioner needed to consistently move an object mimicking the MRI contrast properties of tissue, also called a phantom, to designated positions with micrometer-scale precision. The movements also needed to be synchronized with MEG pulses generated by the MR-ARFI pulse sequences. To simulate ARFI and to keep echo times reasonably short for signal acquisition, the micropositioner needed to move the phantom up to ${20}\,{\upmu }\text {m}$ in less than 5 ms. No ferromagnetic materials could be used for any components that would be inside the room with the MRI scanner, which precluded the use of conventional linear actuators, stepper motors, and servos. Metallic components were minimized to reduce susceptibility artifacts, potential motion caused by eddy currents, and the risk of device heating. The device could not produce electromagnetic interference (EMI) that would cause imaging artifacts, and radio frequency pulses generated by the MRI scanner could not significantly affect positioner performance.

## Methods

### Micropositioner

**Fig. 1:**
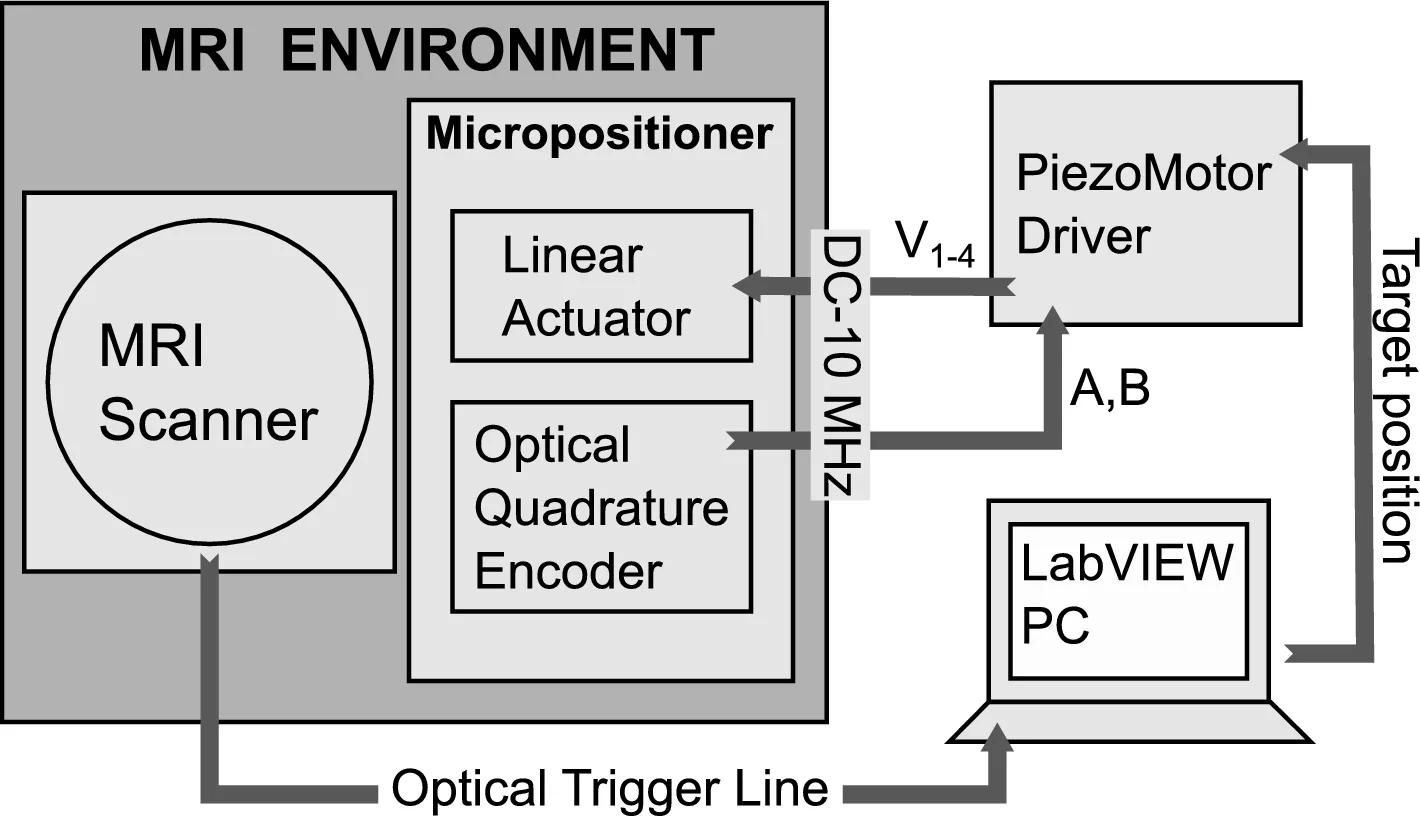
Block diagram of the micropositioning system. The MRI scanner sends an optical trigger signal that is converted to a TTL pulse and received by the PC. A LabVIEW program sends the target position to the motor driver. The driver sends four voltage waveforms $V_{1-4}$ to the piezoelectric linear actuator to produce microsteps. Quadrature signals A,B were sent from the optical encoder to the motor driver for closed-loop control. Electrical signals passed to and from the MRI environment were filtered to attenuate frequency components greater than 10 MHz

Figure 1 shows a block diagram of the major components of the micropositioning system. For compatibility with the MR environment, we chose a nonmagnetic, piezoelectric linear actuator (LT20C, PiezoMotor, Uppsala, Sweden). A transmissive optical encoder module (EM2, US Digital, Vancouver, USA) with digital A and B quadrature outputs was used for closed-loop control of the linear actuator. A linear encoder strip with 2000 lines per inch (LIN-2000-3.5-0.5) gave a resolution of ${3.1}\,{\upmu }\text {m}$ per encoder count. For EMI protection, shielded cables delivered signals between the motor driver (PMD101, PiezoMotor, Uppsala, Sweden), the actuator, and the encoder module. These signals were filtered at the penetration panel of the MRI suite to attenuate frequency components greater than 10 MHz that may have interfered with imaging or device function.

**Fig. 2:**
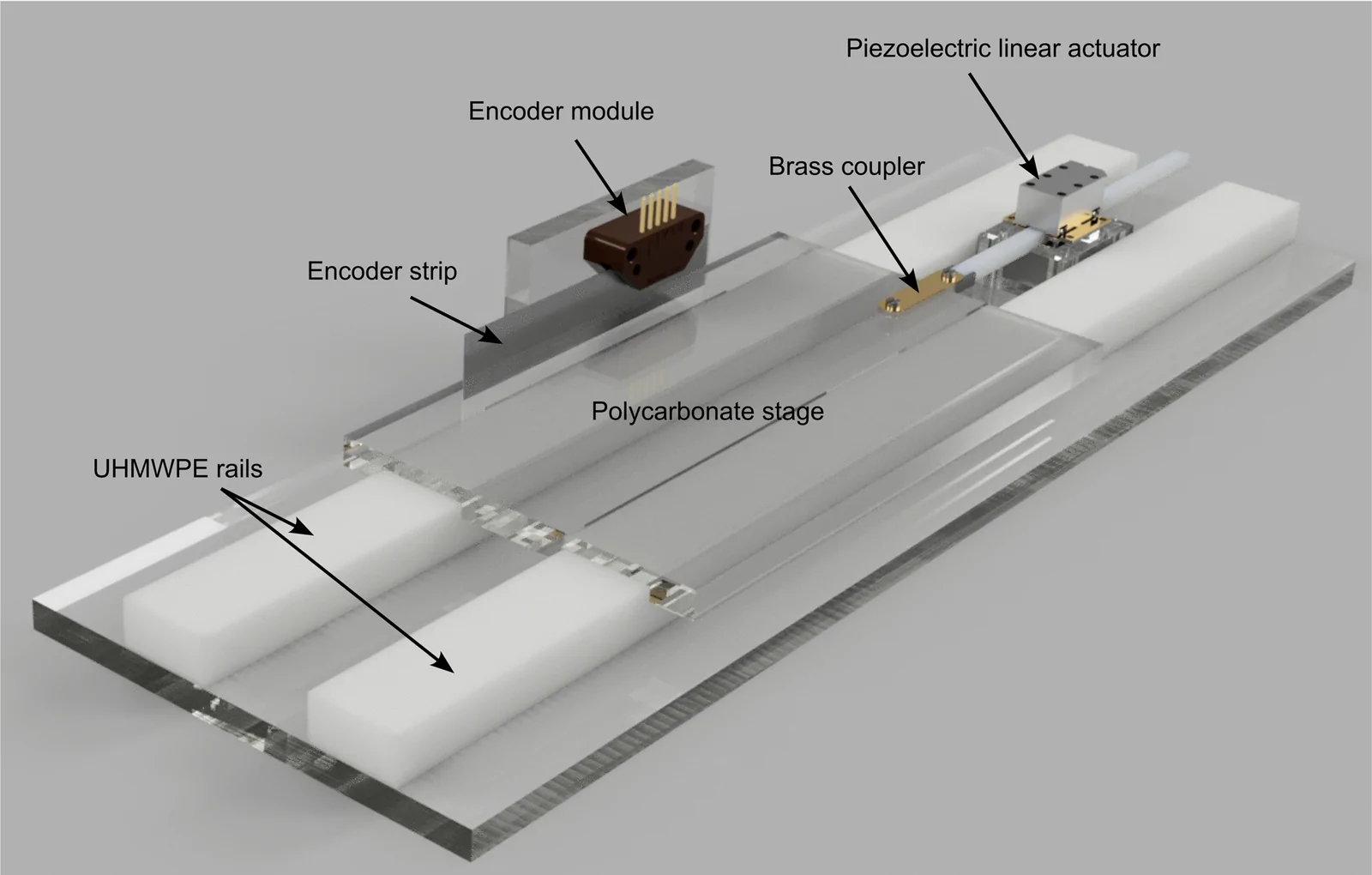
Rendering of micropositioner. Motion was constrained to one dimension using UHMWPE rails. A brass coupler minimized transverse stresses on the piezoelectric actuator’s drive rod. A linear optical encoder strip with 2000 lines/inch was epoxied to the polycarbonate stage and was read by a quadrature encoder module to provide independent displacement measurements for closed-loop actuation

**Fig. 3:**
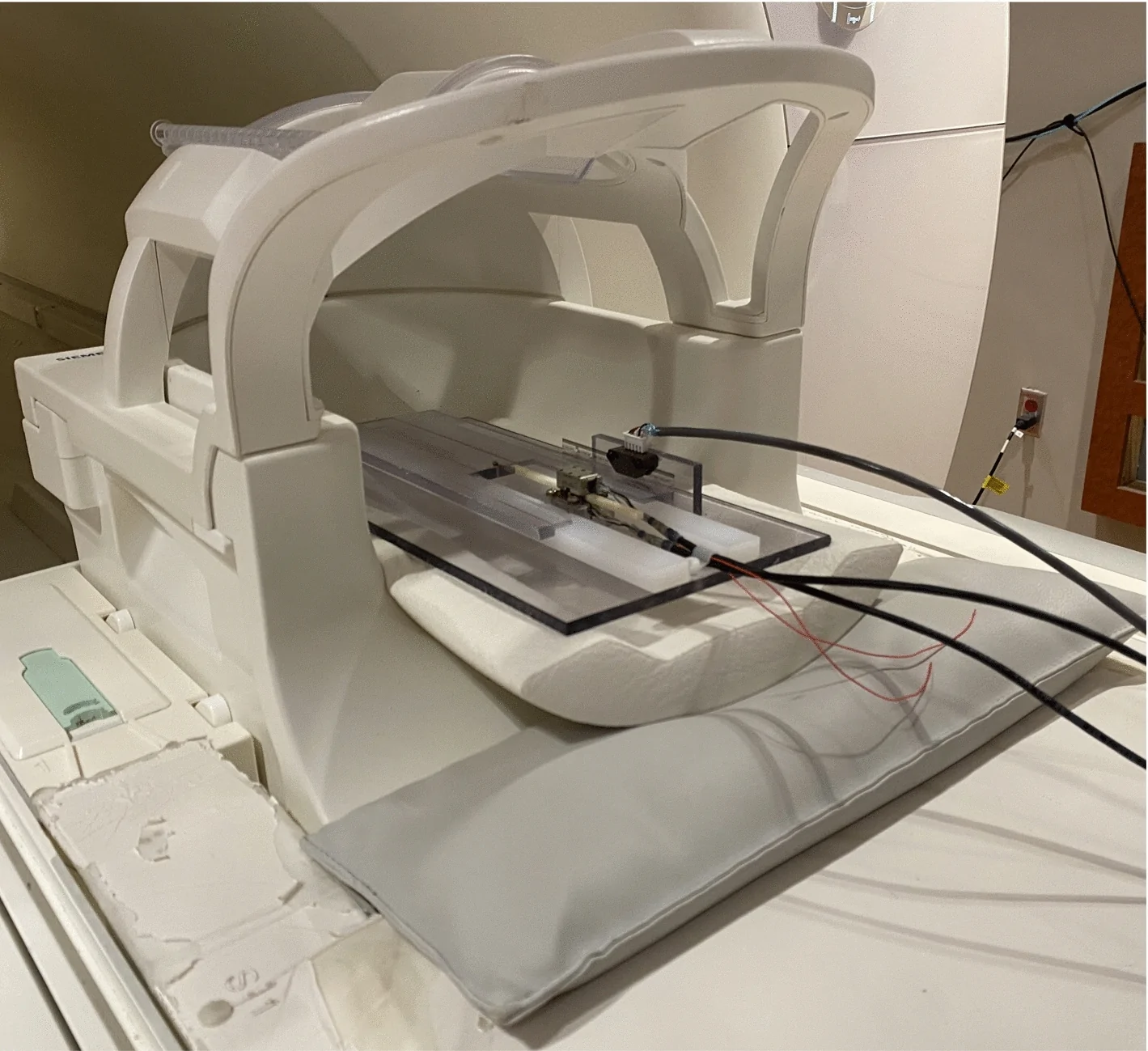
Photograph of the experimental environment. MRI scanner: 3T MAGNETOM Prisma (Siemens Healthineers). Receive coil: 20-channel head and neck. Motion axis was aligned to the MRI bore axis using the scanner’s laser positioning markers. A bubble level was used to ensure the base plate of the micropositioner was parallel to the horizontal plane. Ambient temperature: ${20}^{\,\circ }$C

Figure 2 shows a rendering of the micropositioner. Two parallel guide rails were milled from ultra-high-molecular-weight polyethylene (UHMWPE). UHMWPE is frequently used in industrial applications like slide bearings and was selected for its dimensional stability, abrasion resistance, low coefficient of friction, and low cost compared to other nonmetallic materials. To constrain sample motion to one dimension, grooves matching the width and spacing of the guide rails were milled into an 8-cm × 13-cm rectangular polycarbonate stage. The stage was coupled to one end of the linear actuator’s drive rod using a brass coupler (15 mm × 2 mm × 0.1 mm) and M1.6 PEEK screws. This configuration, recommended by the manufacturer of the linear actuator, prevented excessive nonaxial loads on the drive rod that may have caused piezoelectric motor components to lose contact with the drive rod.

The motor driver was controlled via a LabView VI (NI, Austin, TX, USA). An optical trigger pulse was output from the MRI scanner by the pulse sequence. The offset between the start of the trigger pulse and the start of the MEG lobe could be set on the scanner console.

### Agar phantoms

For the experiments, a gel phantom was selected over a fluid phantom to minimize inertial flow that would introduce confounding phase shifts and signal loss. To prepare the phantom, 3 g of granulated agar was added to 150 mL of deionized water. The mixture was heated and stirred constantly until it boiled for several seconds. To shorten the longitudinal relaxation time (T_1_) and more closely approximate that of tissue, 2 mL of 40 mM CuSO4 solution was added to 100 g of the mixture. This was poured into a cubic mold and cooled to room temperature. The final dimensions of the agar gel were 5 cm × 5 cm × 4 cm. The agar gel was wrapped in plastic wrap and was secured to the micropositioner stage using double-sided tape.

### Magnetic resonance imaging

MRI was performed on a MAGNETOM Prisma 3-tesla scanner (Siemens Healthineers, Erlangen, Germany). Figure 3 shows the experimental setup. The micropositioner was placed on a foam leveling pad inside a head-and-neck coil array with 20 receiver channels. The motion axis of the micropositioner was centered and aligned with the bore axis of the scanner using laser guides. The center of the phantom was then placed at the isocenter of the MRI scanner. Phantom and bore temperatures were ${20}^{\,\circ }$C.

#### Pulse sequences

**Table 1:** MR-ARFI pulse sequence parameters

Sequence	TR/ms	TE/ms	FA/deg	Voxel size/$\textrm{mm}^3$	Acq. time
GRE-ARFI	50	34	15	$1\times 1\times 5$	13s
GRE-EPI-ARFI	500	50	20	$2\times 2\times 5$	18s
SE-ARFI	500	50	90	$2\times 2\times 5$	64s

MEG duration and amplitude were fixed at 10 ms and 30 mT/m, respectively, across all sequences

*TR* repetition time, *TE* echo time, *FA* flip angle, *MEG* motion-encoding gradient, *Voxel size* readout × phase encoding × slice, *Acq. time* acquisition time

**Fig. 4:**
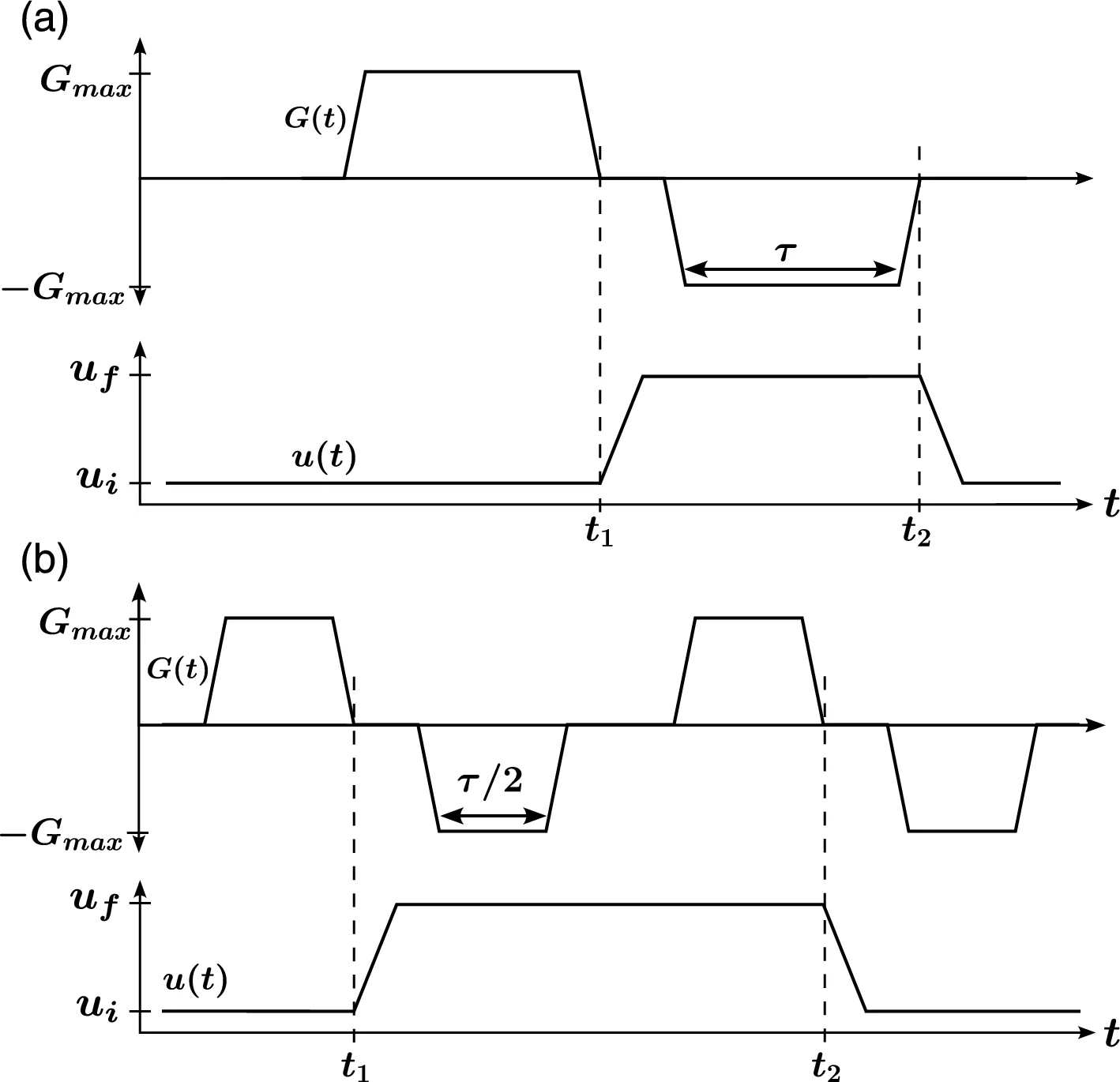
MRI motion-encoding schemes used for **a** GRE- and GRE-EPI-ARFI and **b** SE-ARFI. *G*(*t*) is the MEG waveform. τ is the encoding time of the MEGs. Imaging gradients are not shown. *u*(*t*) is the position of the micropositioning stage. $t_1$ and $t_2$ are times the optical trigger pulses are sent from the MRI scanner. The stage is initially at position $u_i$. The stage is triggered to extend to $u_f$ at $t_1$. The stage is triggered to retract to $u_i$ at $t_2$

Three MR-ARFI pulse sequences were used to measure phase shifts associated with known displacements. Pulse sequence parameters are given in Table 1. The maximum MEG amplitude was ${30}\,\mathrm{mT/m}$. The duration of the constant part of each MEG lobe was 10 ms for the GRE and GRE-EPI sequences and 5 ms for the SE sequence. The MEG slew rate was ${100}\,\mathrm{mT/(m ms)}$, and the corresponding ramp times were ${300}\,{\upmu }\text {s}$. The motion-encoding schemes used in the pulse sequences are shown in Fig. 4. The top plot of each figure shows the MEG amplitude as a function of time, and the bottom plot shows the position of the micropositioner stage *u*(*t*). For clarity, the imaging gradients are not shown in the top part of the figures. The dashed vertical lines in Fig. 4 at times $t_1$ and $t_2$ represent the rising edges of the optical trigger signals from the MRI scanner.

Monopolar MEG lobes of opposite polarity were played between the excitation pulse and readout of a standard 2D GRE pulse sequence, as shown in Fig. 4a. The same encoding scheme was used in a GRE-EPI pulse sequence. The GRE-EPI-ARFI sequence employed a segmented EPI readout with an EPI factor of 7, enabling faster acquisition at the expense of increased geometric distortion and lower SNR. The other sequences used conventional gradient or spin echo readouts. Details of the EPI-ARFI implementation are given in Ref. [9]. For the spin echo sequence, bipolar MEGs were added to either side of the ${180}^{\circ }$ refocusing pulse, as shown in Fig. 4b. The ${180}^{\circ }$ pulse was centered between $t_1$ and $t_2$, so no net phase would accumulate during the imaging gradient associated with this pulse.

Based on motor speed, the triggers were set to fire 3 ms before the initial ramp of the second MEG lobe in the GRE-ARFI and GRE-EPI-ARFI pulse sequences. SE-ARFI used bipolar MEGs, which were repeated with the same polarity after the refocusing RF pulse. The micropositioner was triggered to move to its extended position $u_f$ 3 ms before the second lobe of the first pulse and to move back to its retracted position $u_i$ 3 ms before the second lobe of the second pulse. The dotted vertical lines at times $t_1$ and $t_2$ in Fig. 4 depict the rising edges of the trigger pulses from the MRI scanner. Gaps lasting 3 ms were added between each MEG lobe in each MR-ARFI pulse sequence to allow the stage to reach its extended position before motion encoding began. Similar allowances are made in ultrasound-driven MR-ARFI implementations for the material to reach mechanical equilibrium. For each acquisition, the target encoder count for the extended position of the stage was set to between -5 and 5, corresponding to $u_f\in \{{0}\,{\upmu }\text {m}, \pm {3.2}\,{\upmu }\text {m}, \pm {6.3}\,{\upmu }\text {m}, \pm {9.5}\,{\upmu }\text {m}, \pm {12.7}\,{\upmu }\text {m}, \pm {15.9}\,{\upmu }\text {m}\}$.

#### Image reconstruction

Raw k-space data from the MRI scanner was converted to ISMRM raw data format for processing [17]. Images were reconstructed using the fft function in MATLAB (R2024a, MathWorks, Natick, USA). Individual reconstructed images from each receiver channel were combined using the method of Roemer et al. [18, Eq. 20]. Phase shift images were calculated as $\Delta \phi = \angle {S^*_0 S_f}$, where $S_0$ was the reconstructed complex MR image acquired without triggers such that stage displacement was zero, and $S_D$ was acquired with triggers such that stage displacement was $u_f - u_i$.

#### Image quality

To evaluate the effect of the micropositioner on overall image quality, images were acquired with and without the micropositioner in the MRI environment. With the instrument in the MRI scanner, images were also acquired with the motor and encoder in powered and unpowered states. Signal-to-noise ratios (SNR) were calculated from magnitude images acquired using the three MR-ARFI pulse sequences as well as a magnetization-prepared rapid gradient echo (MPRAGE) pulse sequence that is commonly used for anatomical brain imaging. Magnitude images were thresholded using Otsu’s method, then signal and background regions were segmented based on whether the image intensity was greater than or less than the threshold intensity value, respectively [19]. SNR was calculated as the ratio of the mean intensity value in the signal region to the standard deviation (SD) of intensity values in the background region. For phase images acquired with the three ARFI pulse sequences, a 1 cm × 2 cm region of interest (ROI) was defined in each set of phase shift images. The SD of phase values in the ROI were used to assess the effect of the instrument on the precision of MR phase measurements.

#### Statistical analysis

The overall accuracy of MR-ARFI displacement measurements in the ROI was summarized using the root mean squared error (RMSE) calculated using the following formula:

2 $$\begin{aligned} \text {RMSE}=\frac{1}{n}\sqrt{\sum _{j=1}^{n}\left| u_j-\hat{u}_j\right| ^2}\times 100\%, \end{aligned}$$

where *n* was the number of phase shift images that were acquired for a given pulse sequence, $u_j$ were the mean displacement values calculated in the ROI, and $\hat{u}_j$ were the displacement values recorded by the encoder.

The linearity of MR-ARFI measurements was evaluated with the coefficients of determination $R^2$ from linear regressions between measured phase shifts and encoded displacements. The statistical significance of proportional measurement bias was evaluated using two-tailed t-tests between the slope of each linear regression and the proportionality factor $\gamma G \tau$.

Bland–Altman analysis was performed to assess bias, variability and trends in the MRI measurements with respect to displacement magnitudes. Differences between the MR-ARFI estimated displacements and the actual displacement value determined by encoder readings were plotted against mean values of the two quantities. Limits of agreement were calculated as the overall mean difference for a particular pulse sequence ± 1.96 standard deviations in the differences.

## Results

### Benchtop characterization

**Fig. 5:**
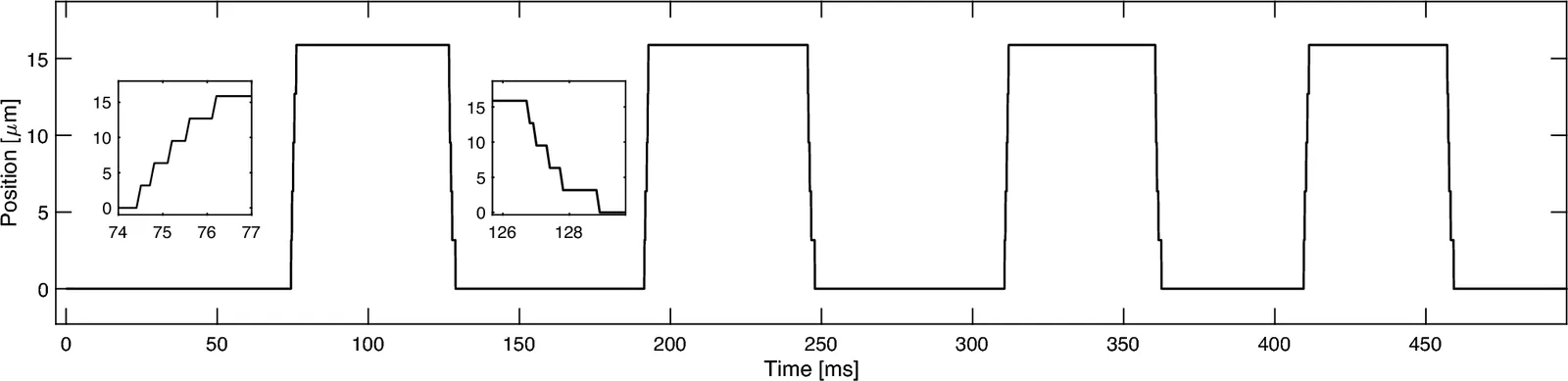
Position vs. time data recorded from encoder module with sample stage loaded with a gel phantom. The sample rate was 10 samples per millisecond. Positive and negative displacement of the stage showed similar speeds of ≈ 7 µm/ms. Insets show zoomed in views of positive and negative displacement performance

Motor speed under closed-loop control was measured by sampling encoder counts from a 32-bit CMOS quadrature counter (LS7366R, LSI Computer Systems, Melville, NY) at 10 kHz using an ATmega328P-based microcontroller board (Arduino). Figure 5 shows micropositioner performance under experimental conditions with a 100-gram gel phantom loaded on the stage. The micropositioner was programmed to move between baseline position $u_i= {0}\,{\upmu }\text {m}$ and target extension $u_f= {15.9}\,{\upmu }\text {m}$ upon receiving a TTL trigger pulse. Trigger pulses were manually generated using keypresses in the LabView VI. Four extension–retraction cycles are shown in Fig. 5. The small insets show positive and negative displacement performance at shorter time scales. The discrete steps in the curves result from the ${3.1}\,{\upmu }\text {m}$ encoder resolution. Typical times to reach target extension were 1.6 ± 0.4 ms, and typical times to return to baseline position were 2.2 ± 0.2 ms. These results informed MR pulse sequence design to ensure that phantoms were static during MR-ARFI MEG pulses.

### MR-ARFI phase shift images

**Fig. 6:**
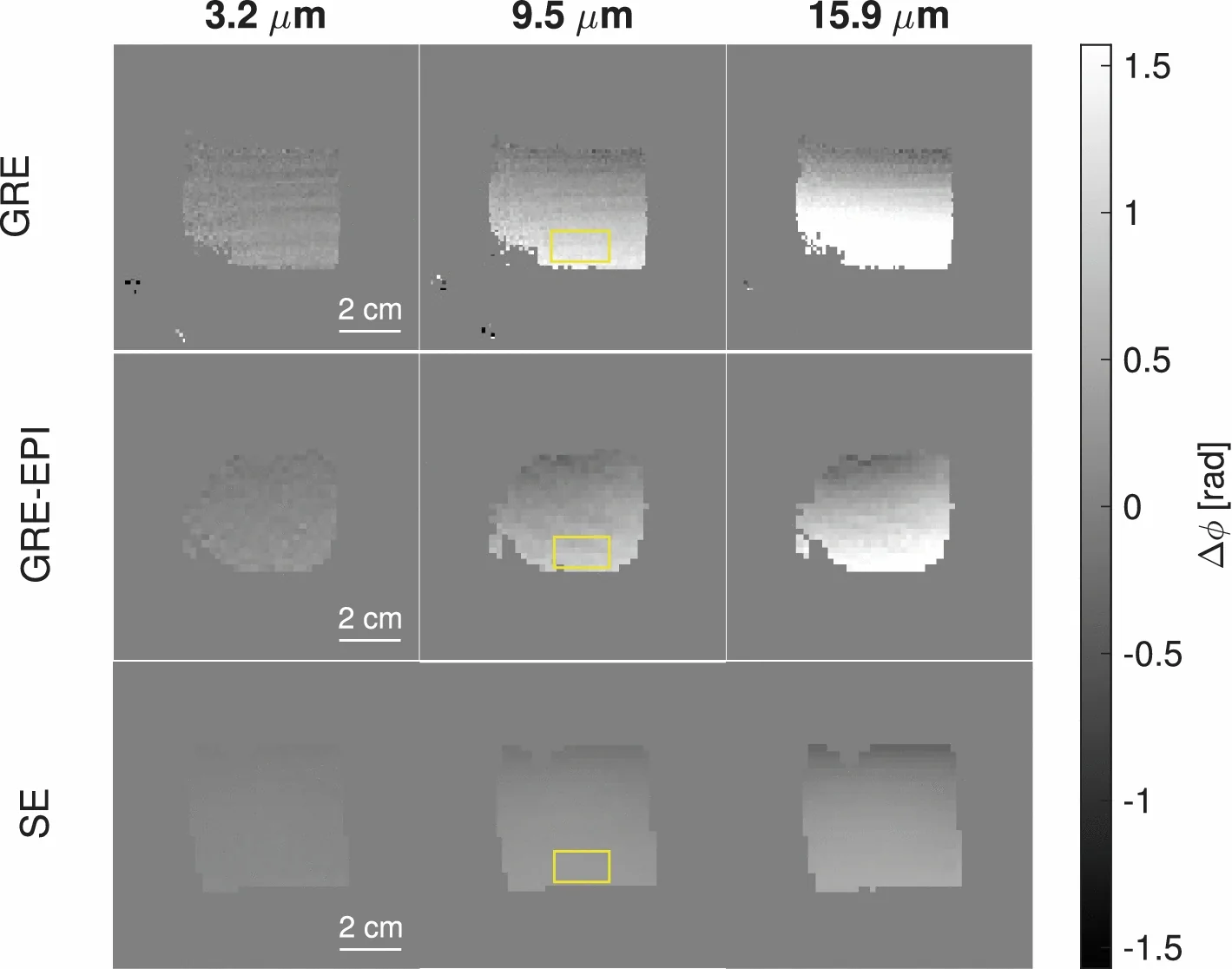
Representative phase shift images calculated from MR-ARFI acquisitions. Phase shift images were calculated as $\Delta \phi = \angle {S^*_i S_f}$, where $S_i$ was the reconstructed complex MR image acquired when the phantom was not moved from $u_i$ and $S_f$ was acquired when it was moved to $u_f$ for displacement encoding. Vertical phase shift gradients were due to the finite shear wave speed in the elastic phantom. Yellow boxes: 1-cm × 2-cm regions of interest used for subsequent analyses

Figure 6 shows phase shift images acquired from each MR-ARFI pulse sequence. Images in each row were acquired using the pulse sequence listed to the left of each row. Target encoder displacement values $\Delta u=u_f-u_i$ used for acquisitions are at the top of each column. For clarity, a binary mask was applied to the phase shift images. A global intensity threshold for the phantom volume was calculated from reconstructed magnitude images using Otsu’s method implemented in MATLAB’s graythresh function [19]. The yellow boxes outline the ROIs used for subsequent analyses. Qualitatively, the GRE and SE images looked similar, but geometric distortions were apparent in the GRE-EPI images. These distortions commonly arise in EPI acquisitions around discontinuities in magnetic susceptibility at material interfaces. Eddy currents induced in metallic components of the scanner or the instrument may have also contributed to some phase effects in GRE and EPI sequences.

The maximum phase shifts measured by the GRE, GRE-EPI, and SE MR-ARFI pulse sequences in the ROI were 2.64, 1.62, and 0.55 radians, respectively, corresponding to the maximum displacement of ${15.9}\,{\upmu }\text {m}$. The minimum phase shifts were −2.13, −1.37, and 0.64 radians, corresponding to displacements of ${-12.7}\,{\upmu }\text {m}$, ${-15.9}\,{\upmu }\text {m}$, and ${-15.9}\,{\upmu }\text {m}$, respectively. The mean magnitude of phase shifts measured by the GRE and GRE-EPI sequences was about two times greater than those measured by the SE sequence.

Vertical phase gradients appeared in all of the phase shift images for displacements greater than zero. The phase gradients were approximately linear and were likely artifacts from the experimental phantom setup. Since the agar phantom was an elastic material, and since the sides of the phantom were not enclosed in a rigid material, only the bottom face of the phantom that was secured to the stage would move nearly instantaneously after the initial trigger. The rest of the phantom would move only when the induced shear wave reached that part of the material. In a previous study, the shear wave speed of 2% agar gels was found to be around 3.6 m/s [20]. Displacement was encoded during the interval from 3 ms to 13 ms after the initial trigger. The shear wave would be expected to reach the top of the phantom approximately 11 ms after the trigger. Reflected waves would therefore return to reach the bottom of the phantom around 11 ms later, after the encoding period had ended, so the MR phase measurements in the ROI near the bottom of the gel were not expected to be significantly affected by the reflections.

### Effects of the micropositioner on image quality

**Fig. 7:**
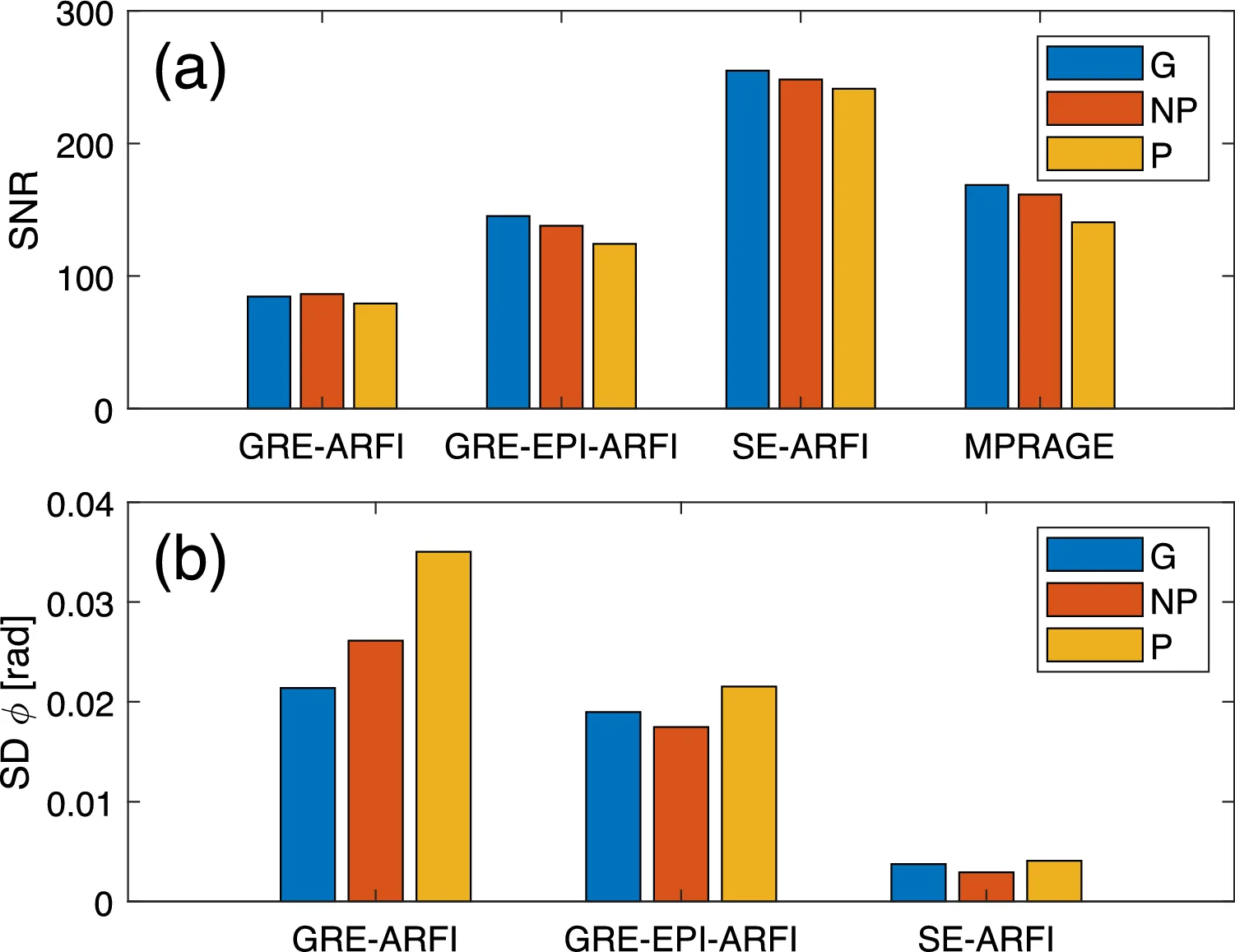
Impact of micropositioner on image quality and phase precision. **a** Signal-to-noise ratios of MR-ARFI and MPRAGE pulse sequences measured in gel phantom without the micropositioner in the MRI scanner (G, blue bars, left), with the micropositioner and gel set up in the scanner but left unpowered (NP, orange bars, middle), and with the micropositioner plugged in and the motor energized (P, yellow bars, right). In the powered state, no triggers were processed and the gel phantom was stationary for all acquisitions. **b** Standard deviations of phase values in the ROI of MR-ARFI phase images

The bar graph in Fig. 7a shows the SNR of magnitude images acquired with the three MR-ARFI sequences and an MPRAGE sequence that is typically used for anatomical imaging of the brain. SNR values depicted with blue bars on the left of each group were calculated from images of the gel phantom that were acquired when the micropositioner was outside of the MRI environment. The orange bars in the center of each group represent SNR values that were calculated from images acquired with the micropositioner in place but with none of the connections made at the penetration panel of the RF shield. The yellow bars on the right side of each group show SNR values calculated from images acquired with the micropositioner in place and fully powered. The stage was kept stationary during all of the acquisitions.

The SE-ARFI pulse sequence produced images with the highest overall SNR. SNR decreased by 2.5% when the micropositioner was set up with the phantom in its unpowered state and decreased by another 2.8% when the instrument was powered. The SNR of images acquired with the GRE-EPI-ARFI sequence was about 43% lower than the SE-ARFI images. For this sequence, SNR decreased by 5% upon addition of the micropositioner to the scanner and decreased by another 10% after applying power. The SNR of GRE-ARFI images was about 68% lower than the SE-ARFI images, which was consistent with the expected SNR decrease based on the relative voxel volumes. The SNR decreased by about 1% with the unpowered micropositioner, and powering the device lowered the SNR by an additional 3%.

Figure 7b shows the phase SD values within the ROIs of phase images acquired with each of the MR-ARFI pulse sequences. Without the micropositioner, the GRE-ARFI sequence showed the lowest phase precision with SD of 0.02 radians, 13% higher than the GRE-EPI-ARFI sequence and four times higher than the SE-ARFI sequence. After the micropositioner was added to the imaging setup, the phase SD for GRE-ARFI increased by 22% to 0.026 radians. However, the phase SD for GRE-EPI-ARFI decreased by 8% to 0.018 radians, and the phase SD for SE-ARFI decreased by 22% to 0.0029 radians. When the micropositioner was powered on, the phase SD of GRE-ARFI increased by 34% to 0.035 radians, GRE-EPI-ARFI increased by 23% to 0.022 radians, and SE-ARFI increased by 40% to 0.0041 radians, relative to the unpowered condition.

### Accuracy and linearity of encoded phase shifts

**Fig. 8:**
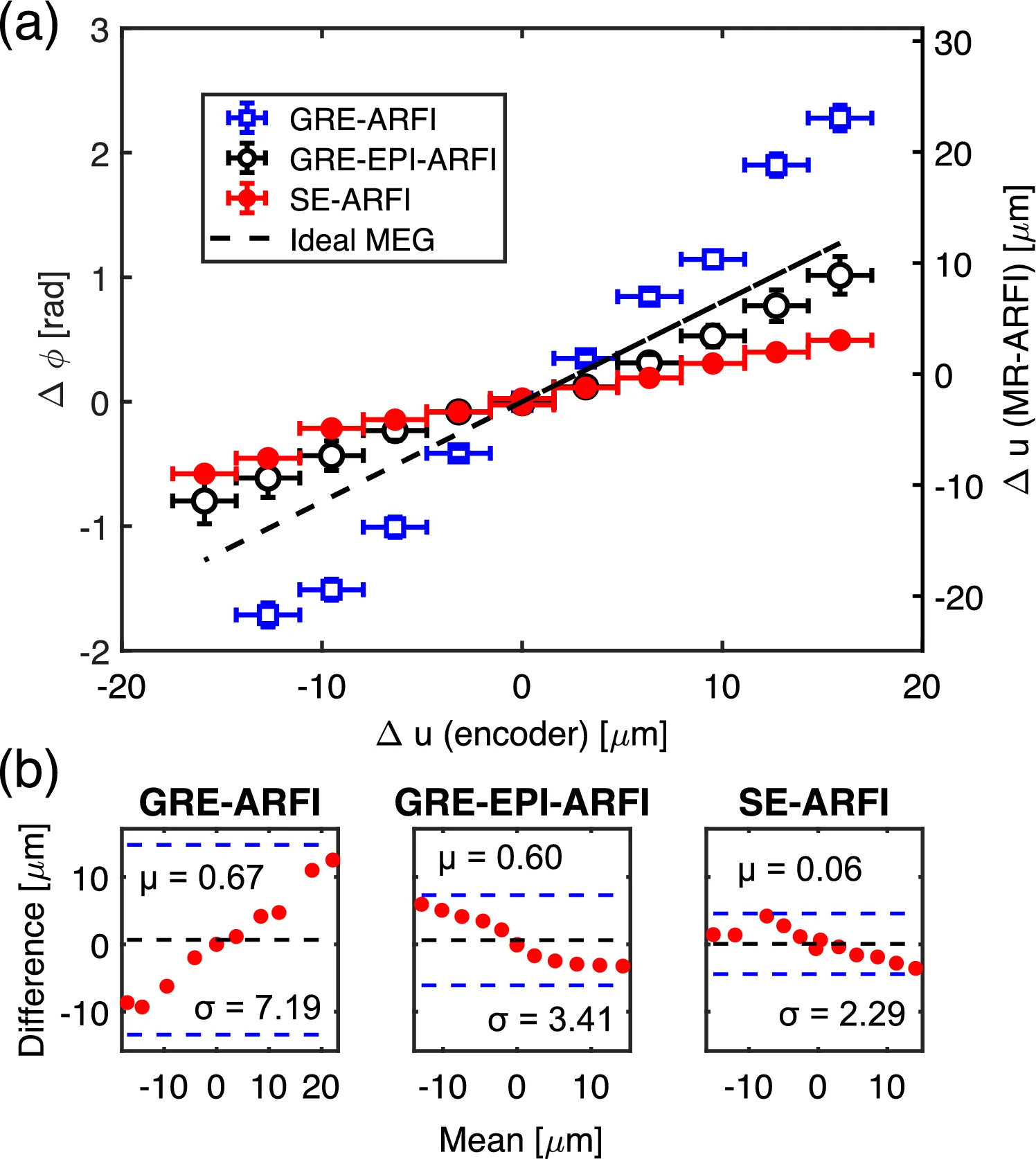
Accuracy and bias of MR-ARFI displacement measurements. **a** Mean MR phase shift $\Delta \phi$ versus displacement Δu measured with encoder. Right vertical axis shows the corresponding Δu values based on $\Delta \phi$ and Eq. (1). Lengths of vertical error bars correspond to standard deviations of phase shifts inside regions of interest (ROIs) shown in Figure 6. Length of horizontal error bars corresponds to encoder resolution and step size of linear actuator. Dashed line is the linear relationship between phase shift $\Delta \phi$ and displacement assuming ideal, rectangular MEG with amplitude $G_{\text {max}}=$ 30 mT/m and duration τ= 10 ms. **b** Bland–Altman plots of the difference between MR-ARFI displacement measurements based on phase shifts Eq. (1) and encoder measurements versus the mean of the two measurements. Black dashed lines: mean difference values. Blue dashed lines: limits of agreement calculated as mean difference ± 1.96 standard deviations of difference values

Figure 8a plots the mean phase shifts in the ROIs at the target displacements. The ROIs are shown in Fig. 6. The length of each vertical error bar is the standard deviation of the phase shifts in the ROI of a particular displacement image, and the length of each horizontal error bar is the encoder resolution. The dashed line is the theoretical linear relationship between phase shift and displacement, given in Eq. (1), assuming a rectangular gradient with amplitude $G_\textrm{max}=$ 30 mT/m and duration τ= 10 ms. Root mean squared error values for mean displacements calculated within the ROIs for the GRE, GRE-EPI, and SE MR-ARFI pulse sequences were 6.9, 3.3, and $5.8 \,{\upmu }\text {m}$, respectively.

If the ramp times for the gradients are taken into account, the area of each MEG lobe, and the resulting phase sensitivity of the GRE sequences increases by 3%. For the SE sequence, the MEG area and sensitivity increase by 6% because twice as many gradient lobes were used for displacement encoding. In this case, the RMSE for GRE, GRE-EPI, and SE sequences were 6.4, 3.5, and $6.0 \,{\upmu }\text {m}$, respectively.

The linearity of each measurement was evaluated using a linear regression between the mean measured phase shifts in the ROI and the encoded displacements. The resulting $R^2$ values for the GRE, GRE-EPI, and SE sequences were 0.994, 0.987, and 0.982, respectively. These values indicate reasonably linear measurements; however, the slope of each regression was significantly different than the theoretical proportionality factor in Eq. (1) at a significance level of 0.1%.

### Bias of encoded phase shifts

Bland–Altman analysis was used to evaluate trends and bias in displacement encoding up to ${15}\,{\upmu }\text {m}$. Figure 8b shows Bland–Altman plots for the three pulse sequences. The differences between MR-ARFI and encoder measurements evaluated in the ROIs are plotted against the mean of the two values for each displacement. The black dashed line in the plots is the mean difference value, and the blue dashed lines are the upper and lower limits of agreement, which were calculated as the mean difference ± 1.96 standard deviations of the difference values. The mean differences were 0.67, 0.60, and $0.06 \,{\upmu }\text {m}$ for the GRE, GRE-EPI, and SE sequences, respectively. The standard deviations of the differences were 7.19, 3.14, and $2.29 \,{\upmu }\text {m}$. Measurements without scaling biases would lie along a horizontal line, indicating that the displacement magnitude has no effect on the accuracy of the measurement. Each pulse sequence displayed some scaling bias. GRE-ARFI and SE-ARFI measurements showed approximately linear scaling biases, with the exception of outlier SE-ARFI measurements at ${-15.9}\,{\upmu }\text {m}$ and ${-12.7}\,{\upmu }\text {m}$. The biases for the two sequences had slopes of opposite signs, indicating that GRE measurements systematically overestimated displacement magnitude, while SE measurements systematically underestimated displacement magnitude. GRE-EPI-ARFI measurements showed systematic underestimation of displacement magnitude with nonmonotonic scaling bias behavior. GRE-EPI-ARFI measurements approached a maximum absolute deviation of ${5}\,{\upmu }\text {m}$ from encoder measurements at large displacements.

## Discussion

The MRI-compatible micropositioner presented here can serve as a valuable tool to validate the displacement estimates of MR-ARFI pulse sequences optimized for greater sensitivity and reduced scan times. The instrument can also measure inter-scanner variability of MR displacement-encoding techniques. Intrascanner variability due to off-center patient positioning and routine variations in hardware performance can also be assessed. The instrument may also validate velocity and acceleration encoding in MR elastography (MRE) pulse sequences [21, 22], possibly improving the repeatability and accuracy of MRE.

The micropositioner had a minor negative impact on the SNR of MR images acquired with various pulse sequences, including MR-ARFI sequences. The largest decreases in SNR were observed when the powered instrument was placed inside the imager. The observed SNR decreases were likely due to increases in electronic noise transmitted through the shielded cables that carried signals between the micropositioner and the motor controller. The instrument also had an effect on phase precision. Including the micropositioner in the imaging setup resulted in higher phase variance for the GRE-ARFI and SE-ARFI sequences.

We found that three different implementations of MR-ARFI, under ideal conditions with the imaging volume centered in the MRI scanner, demonstrated significant differences in displacement values in terms of accuracy, linearity, and bias. GRE-ARFI and GRE-EPI-ARFI showed opposite trends in bias relative to the theoretical phase shift and mean difference between measured and actual values. Both GRE pulse sequences used the same motion-encoding scheme but different timing parameters and readouts. GRE-ARFI overestimated displacement magnitude, while GRE-EPI-ARFI underestimated displacement magnitude in both directions. This discrepancy could have been due to differences in timing parameters between the two pulse sequences.

It is worth noting that the GRE sequence with the shortest repetition time (TR) and shortest time between displacements showed the largest errors in measured mean displacements. This could have been caused by additional overshoots and undershoots of target position as the allowed time between movements was decreased. Another possibility is that the shorter timing intervals set up a mechanical steady state that skewed the displacement measurements in the opposite direction as the other pulse sequences with ten times longer TR. Finally, the GRE sequence was acquired with four times smaller voxel volumes, so the MR-ARFI encoded displacements would have shown fewer partial volume effects and lower SNR than the sequences acquired with larger voxels.

GRE-ARFI demonstrated high linearity of displacement measurements with $R^2=0.994$, but suffered systematic bias that increased to more than 100% at large displacements. The GRE-EPI pulse sequence produced measurements with lower linearity with $R^2=0.987$ and lower systematic bias of about 50% at higher displacements. The SE pulse sequence showed the lowest linearity with $R^2=0.982$ and the same levels of systematic bias as the GRE-EPI sequence, but with a discontinuity for the two largest negative displacements where the difference between MR-ARFI estimates and encoder measurements was much closer to zero, as shown in Fig. 8b.

MR-ARFI is particularly sensitive to other sources of motion that do not arise from acoustic radiation forces. Much of the work on MR-ARFI has been motivated by the need for transcranial ultrasound targeting and treatment evaluation in the brain [23, 24]. The brain is not as prone to movement during imaging sessions as other organs, especially those in the abdomen, including the liver, which undergo significant displacements during the respiratory cycle. The utility of MR-ARFI in other anatomical regions will depend on the ability to compensate or correct for bulk tissue motion. We expect this instrument to be useful for the development of respiratory gating and motion correction algorithms for MR-ARFI pulse sequences.

Another possible application for calibrated MR-ARFI is to ensure the safety and reliability of medical ultrasound devices. Engineers use testing techniques like the radiation force balance and hydrophone scans in a water tank to characterize the acoustic output of ultrasound transducers. The radiation force balance detects millinewton acoustic forces incident on an absorbing or reflecting target and provides an average measurement over the transducer geometry and the time period over which the measurement is taken. Acoustic pressure field mapping in a testing tank with a hydrophone is a relatively slow process. Detailed three-dimensional maps take days to acquire as the hydrophone is raster scanned point-by-point, but they provide valuable volumetric data engineers and regulatory agencies need to ensure patient safety. However, these techniques map the free-field acoustic device output in water, and the actual effect on tissue must be estimated numerically. Properly calibrated MR-ARFI would be suitable for ultrasound device field mapping in viscoelastic materials, like tissue mimicking phantoms, allowing for rapid 3D field mapping in situ instead of in the free field. Complex calculations of ultrasound bioeffects from free field complex pressure amplitudes would be replaced by direct mapping of physical parameters that are more readily converted to bioeffect magnitude.

As developments in focused ultrasound transducers and techniques lead to innovative new approaches to noninvasive therapeutics, the instrumentation presented here may find use for in situ calibration of MR-ARFI for applications that demand precise and accurate displacement measurements. The main $B_0$ field of MRI scanners is known to drift over minutes [25], introducing phase errors that could manifest in displacement maps despite phase image subtraction. A compact micropositioner could be designed to fit alongside the imaging subject within the field of view to accommodate concurrent calibrated MR-ARFI. In this process, systematic errors in MR-ARFI displacement estimates could be regressed out to provide more accurate displacement measurements.

The methodology presented here used a micropositioner with only one translational degree of freedom. The most general MR-ARFI measurements are done along three axes. Clinical use cases for MR-ARFI may require measurements and validations along multiple axes. The instrument we implemented could be extended to produce translational motion along multiple axes utilizing piezoelectric positioning systems with multiple degrees of freedom, even rotational. One or multiple positioning systems could also be used to apply known stresses and strains to materials in order to validate measurements of displacement fields with spatial gradients. This would be useful to assess partial volume effects in MR-ARFI acquisitions with different geometric parameters or with RF pulses that produce different slice profiles.

This study had several limitations. First, only one phantom was used, so displacement measurement variability due to material composition and magnetic properties could not be evaluated. Second, repeat measurements were not made, so no conclusions about scan-to-scan repeatability could be drawn. Third, precautions were taken to minimize EMI, but we did not monitor voltage or current in the electrical connections. Any transients induced by the MR imager that may have affected the electronic components of the micropositioner were not detected. Benchtop measurements of motor speed relied on manual software triggers that were 50 to 100 ms apart and may not reflect actual motor performance when repeatedly triggered at shorter intervals used in imaging experiments. The device could be simplified using self-sensing piezoelectric actuation techniques described by Liseli et al [26], reducing latency and set up times. To maximize accuracy, precision, and clinical utility, it will be necessary to perform additional trials with a higher precision reference like a laser interferometer.

## Conclusion

We have successfully designed and implemented a nonmagnetic micropositioner that enables reliable validation of micrometer-scale displacement measurements in the MRI environment. The system, built with MRI-compatible materials and components, demonstrated consistent performance with positioning times of 1.6±0.4 ms for extension and 2.2±0.2 ms for return to baseline of a 100-gram agar phantom. This speed allowed for synchronization with MEG pulses in MR-ARFI sequences. The micropositioner provides a reliable reference standard for evaluating the accuracy of displacement-based measurements across different pulse sequences, including 2D GRE, SE, and GRE-EPI. This instrumentation serves as a valuable tool for calibrating MR-ARFI pulse sequences, potentially improving their quantitative accuracy for applications in treatment dosimetry and biomarker imaging.

## Data Availability

The datasets used in this study are available from the corresponding author upon reasonable request.

## Code availability

The code and scripts used in this study are available from the corresponding author upon reasonable request.
